# Cirrhotic cardiomyopathy

**DOI:** 10.1186/1750-1172-2-15

**Published:** 2007-03-27

**Authors:** Soon Koo Baik, Tamer R Fouad, Samuel S Lee

**Affiliations:** 1 Dept of Medicine, Yonsei University Wonju College of Medicine, Wonju, South Korea; 2 Liver Unit, University of Calgary, Calgary, Canada

## Abstract

Cirrhotic cardiomyopathy is the term used to describe a constellation of features indicative of abnormal heart structure and function in patients with cirrhosis. These include systolic and diastolic dysfunction, electrophysiological changes, and macroscopic and microscopic structural changes. The prevalence of cirrhotic cardiomyopathy remains unknown at present, mostly because the disease is generally latent and shows itself when the patient is subjected to stress such as exercise, drugs, hemorrhage and surgery. The main clinical features of cirrhotic cardiomyopathy include baseline increased cardiac output, attenuated systolic contraction or diastolic relaxation in response to physiologic, pharmacologic and surgical stress, and electrical conductance abnormalities (prolonged QT interval). In the majority of cases, diastolic dysfunction precedes systolic dysfunction, which tends to manifest only under conditions of stress. Generally, cirrhotic cardiomyopathy with overt severe heart failure is rare. Major stresses on the cardiovascular system such as liver transplantation, infections and insertion of transjugular intrahepatic portosystemic stent-shunts (TIPS) can unmask the presence of cirrhotic cardiomyopathy and thereby convert latent to overt heart failure. Cirrhotic cardiomyopathy may also contribute to the pathogenesis of hepatorenal syndrome. Pathogenic mechanisms of cirrhotic cardiomyopathy are multiple and include abnormal membrane biophysical characteristics, impaired **β**-adrenergic receptor signal transduction and increased activity of negative-inotropic pathways mediated by cGMP. Diagnosis and differential diagnosis require a careful assessment of patient history probing for excessive alcohol, physical examination for signs of hypertension such as retinal vascular changes, and appropriate diagnostic tests such as exercise stress electrocardiography, nuclear heart scans and coronary angiography. Current management recommendations include empirical, nonspecific and mainly supportive measures. The exact prognosis remains unclear. The extent of cirrhotic cardiomyopathy generally correlates to the degree of liver insufficiency. Reversibility is possible (either pharmacological or after liver transplantation), but further studies are needed.

## Definition and diagnostic criteria

In cirrhosis, cardiac output increases whereas systemic vascular resistance and arterial pressure decrease [1,2]. Despite the increased basal cardiac output, cardiac response to physiologic or pharmacologic stimuli is known to be subnormal [3-5], a phenomenon called "cirrhotic cardiomyopathy" [6,7]. When first described more than 3 decades ago in cirrhotic patients, cardiac dysfunction was presumed to result from alcoholic cardiotoxicity, specifically a latent alcoholic cardiomyopathy [8-10]. However, during the past 2 decades, it has become clear that blunted ventricular contractility with stress is also present in nonalcoholic patients and animal models of cirrhosis [11-13]. In the 1990s, numerous studies in patients with nonalcoholic cirrhosis conclusively demonstrated that depressed ventricular contractile responses to stimuli are found in *all *forms of cirrhosis [14-19].

In the absence of consensus definitions, the term "cirrhotic cardiomyopathy" is defined at present as: 1) baseline increased cardiac output but blunted ventricular response to stimuli, 2) systolic and/or diastolic dysfunction, 3) absence of overt left ventricular failure at rest, 4) electrophysiological abnormalities including prolonged QT interval on electrocardiography and chronotropic incompetence [7,20-22]. Not all features are required for the diagnosis; for example, only 30–60% of patients show a prolonged QT interval.

## Epidemiology

Without firm diagnostic criteria, the exact prevalence of cirrhotic cardiomyopathy remains unknown. Its estimation is a difficult task as the disease is generally latent and shows itself only when the patient is subjected to stress such as body position changes, exercise, drugs, hemorrhage and surgery. The prevalence of liver cirrhosis is also difficult to estimate, because many persons with compensated cirrhosis do not exhibit signs or symptoms of the disease and because noninvasive studies lack sensitivity to detect cirrhosis at early stages. Moreover there is tremendous geographical variability of cirrhosis worldwide, depending on the prevalence of the causative factors such as viral hepatitis B and C, chronic alcoholism, iron overload, autoimmune liver disease, etc.

However, we believe that at least one feature of cirrhotic cardiomyopathy, such as electrocardiographic QT prolongation or diastolic dysfunction, is present in the majority of patients with cirrhosis who have reached Child-Pugh stage B or C (representing moderately or severely advanced liver failure). Moreover, diastolic dysfunction is probably present in virtually all patients with cirrhotic cardiomyopathy, and simple echocardiographic indices such as the E/A ratio may detect diastolic dysfunction even at rest. Indeed, once cirrhosis has advanced to a moderate stage, with the accumulation of peripheral edema or ascites, it appears that some element of diastolic dysfunction is universally present. For now, tests of diastolic function by echocardiography or dynamic magnetic reasonance cardiac imaging may therefore represent the best available screening test to diagnose the syndrome.

## Clinical features

Many aspects of cardiac contraction have been described to be abnormal in cirrhosis, including histology and structure, chronotropic, systolic and diastolic function, and electrophysiological changes.

Functional and structural changes in the cardiac chambers have been seen in the left heart, rather than the right. Most studies have found dilatation of the left atrium, and hypertrophy or dilatation of the left ventricle. The right atrium and right ventricle generally have normal dimensions and wall thickness in the absence of the uncommon syndrome of portopulmonary hypertension. Histological changes of cirrhotic cardiomyopathy include myocardial fibrosis, subendocardial edema, and nuclear and cytoplasmic vacuolation of cardiomyocytes [reviewed in [3,4,7]. However, these conclusions are based on old autopsy studies in patients with alcoholic cirrhosis, some dating back almost 8 decades. Whether some of these finding may simply reflect mild alcoholic cardiotoxicity remains uncertain.

The chronotropic response to the **β**-adrenergic agonist isoproterenol is attenuated in cirrhotic humans and rats [13,23]. Cardiac response to physical exercise in cirrhotic patients is blunted, with subnormal responses in echocardiographic ejection fraction and contraction time. Moreover, both ventricular systolic and diastolic function are impaired in cirrhosis [3,4,7,22]. Stroke volume and contractile indices are typically normal or even increased at rest. However, under stressful stimuli such as exercise, hemorrhage or drug infusions, cirrhotic patients may show an attenuated systolic function compared to healthy controls [6,14-19]. For example, normal subjects can triple their cardiac output with submaximal exercise; cirrhotic patients do not exceed a doubling of cardiac output with exercise [14]. The prevalence and extent of systolic dysfunction in cirrhotic patients appear to be variable.

In contrast, some element of diastolic dysfunction appears to be more common. Indeed, some authorities contend that some degree of diastolic dysfunction is present in virtually every patient with cirrhosis [16-18,22]. Diastolic dysfunction manifests as a stiff, noncompliant ventricle, and is often seen in patients with some degree of left ventricular hypertrophy or dilatation. However, overt structural changes in the ventricle are not a prerequisite for diastolic dysfunction. Impaired passive and active filling of the left ventricle in early and middle-late diastole, respectively, lead to an inability to adequately increase stroke volume in response to stimuli. Again, in contrast to systolic dysfunction, a significant stimulus may not be required to detect diastolic dysfunction. Echocardiography may reveal abnormal diastolic function even at rest [reviewed in [7,22].

Electrophysiological changes including prolonged repolarization and impaired cardiac excitation-contraction coupling have been demonstrated in cirrhotic patients [24-28]. Repolarization prolongation is manifested by a prolonged QT interval on the electrocardiogram. Rate-corrected prolongation of the QT (>440 msec) is found in 30–60% of patients with cirrhosis [25-28]. Prolongation of QT interval can be associated with an increased risk of certain ventricular arrhythmias, particularly the "torsade de pointes" type of ventricular tachycardia. However, sudden cardiac death is rare in cirrhosis, and torsade de pointes has only been described in a few patients, all of whom had other risk factors for this type of ventricular tachycardia. For example, torsade de pointes has been reported in patients with variceal bleeding receiving vasopressin [29,30]. Therefore, the clinical significance of QT prolongation in cirrhosis remains unclear.

In addition to QT prolongation, Henriksen and colleagues have recently reported that cirrhotic patients show a dispersion of the normal interval between the onset of electrical systole (the electrocardiographic QRS complex) and mechanical systole itself [31]. In other words, this normally tightly-regulated time interval is subject to wider variability in cirrhotic patients, either earlier or later than normal. Again, the clinical relevance of this interesting observation remains unknown at present.

The exact mechanism leading to these electrophysiological changes is unclear. In clinical studies, severity of liver disease and circulatory dysfunction are related to prolonged QT interval. Moreover, these changes disappear after liver transplantation in most patients [25-28]. Studies in the cirrhotic rat suggest that functional alterations of ion channels, particularly one or more types of potassium channels, in cardiac plasma membranes play a role in initiating and maintaining cardiac electrophysiological abnormalities [32,33].

## Clinical consequences

Overt heart failure is rare because of the peripheral vasodilatation characteristic of cirrhosis, in effect "autotreating" the ventricle by systemic vasodilatation reducing afterload, and compensatory diminution of inhibitory influences such as the cardiac muscarinic system [3,34]. Although patients may complain of dyspnoea, reduced exercise capacity, peripheral fluid retention (dependent oedema) and ascites, these symptoms are common to both heart failure and advanced cirrhosis. It is therefore difficult to determine by symptoms if patients are indeed suffering from symptomatic heart failure. The only differentiating feature is that dyspnoea in cirrhosis is usually associated with hydrothorax from ascitic fluid tracking into the pleural cavity or tense ascites pushing up against the respiratory diaphragms, and not with pulmonary vascular congestion or frank pulmonary oedema. The presence of such pulmonary congestion strongly points to a diagnosis of heart failure, whether due to cirrhotic cardiomyopathy or other causes.

Stresses such as liver transplantation, infection and procedures such as insertion of transjugular intrahepatic portosystemic stent-shunts (TIPS) can convert latent to overt heart failure. Indeed, heart failure is responsible for 7–15% of mortality following liver transplantation [5,35], and in some series is the third-leading cause of death after rejection and infection. Numerous studies report acute left ventricular failure or subtler indices of cardiac dysfunction following liver transplantation, even in patients with no previous cardiac history or risk factors [36-40]. For example, 12–56% of patients develop clinically overt or radiographic evidence of pulmonary edema immediately after transplantation, usually within the first postoperative week [22,41].

TIPS are often used to treat recurrent or intractable variceal bleeding, or ascites. The abrupt shift of a significant portion of the mesenteric venous blood directly into the systemic venous circulation through these portosystemic shunts dramatically increases the cardiac preload. Several studies have reported abnormal cardiac response to this abrupt increase in preload [42-44]. There are also several case reports and small series of patients with precipitation of overt left ventricular failure after TIPS insertion [45,46]. Indeed, a recent Spanish-American multicenter study of TIPS *vs *large volume paracentesis for treatment of ascites, reported that 12% of the TIPS group developed heart failure compared to none in the paracentesis group [46]. Recently, Cazzaniga and colleagues [47] demonstrated that the E/A ratio, an indicator of diastolic dysfunction, measured at 4 weeks after TIPS insertion, was the only independent predictor of survival following this procedure. This suggests that cirrhotic cardiomyopathy is a primary determinant of survival in cirrhotic patients undergoing procedures that stress the cardiovascular system [48].

Recent studies suggest that cirrhotic cardiomyopathy may contribute to the pathogenesis of hepatorenal syndrome (HRS) precipitated by spontaneous bacterial peritonitis [49-51]. These Spanish studies demonstrated that amongst patients treated with antibiotics and recovering from spontaneous bacterial peritonitis, hepatorenal syndrome occurred in those whose cardiac output was low at baseline and failed to increased appropriately after infection resolution [49]. Later these authors found that 27 patients of a cohort of 66 cirrhotic patients developed hepatorenal syndrome; cardiac output (lower than in those who did not develop HRS) and plasma renin activity were the only factors predictive of HRS occurrence [50]. The authors suggested that hepatorenal syndrome develops in part due to inadequate cardiac contractility in the face of marked peripheral vasodilatation.

## Etiology and pathogenic mechanisms

There is no direct genetic predisposition to cirrhotic cardiomyopathy. The pathogenic mechanisms of cirrhotic cardiomyopathy include impairment of stimulatory **β**-adrenergic receptor signalling pathways, altered cardiomyocyte plasma membrane physicochemical properties, and overactivity of negative-inotropic factors such as nitric oxide, carbon monoxide and endocannabinoid systems [7,22].

Heart cell contractility is mainly determined by the stimulatory **β**-adrenergic receptor system. When stimulated by norepinephrine or other catecholamines, the **β**-adrenergic receptor-ligand complex couples with G-protein to stimulate membrane-bound adenylate cyclase, generating cAMP which phosphorylates several proteins leading to intracellular calcium fluxes. The calcium then causes actin-myosin cross-linking, and thus cellular contraction [52].

Cardiomyocyte plasma membranes isolated from a rat model of cirrhosis show multiple defects in the **β**-adrenergic signaling pathway and lipid composition of the membrane bilayer [53,54]. Due to increased cholesterol in the membrane, the ratio of cholesterol:phospholipids increases, leading to a biophysical change termed decreased membrane fluidity [53,54]. Decreased membrane fluidity hampers the conformational movement ability and thus the function of protein receptors embedded in the membrane, such as calcium channels and **β**-adrenergic receptors [52].

Other mechanisms include increased inducible nitric oxide synthase (iNOS) activity with overproduction of NO [55-57], and increased heme oxygenase-1 activity that overproduces carbon monoxide [58]. Both gases inhibit cardiomyocyte contractility by stimulating soluble guanylate cyclase to produce cGMP. cGMP inhibits contractility by several mechanisms, mainly by inhibiting calcium release from the sarcoplasmic reticulum of the cardiomyocyte [59].

Myocardial endocannabinoid production may be increased in response to stresses such as increased heart rate and hemodynamic overload [60]; these substances are known to have a negative inotropic effect in rats [61] and humans [62]. In that regard, we have recently demonstrated a pathogenic role of endocannabinoids such as anandamide in rats with biliary cirrhosis [63]. In isolated left ventricular papillary muscles from these cirrhotic rats, increased endocannabinoid signaling *via *CB1 receptors contributes to blunted contractile responsiveness to **β**-adrenergic stimulation [63].

## Diagnostic tests

To date, there is no single diagnostic test that can identify patients with this condition. The heart produces and releases peptide hormones such as natriuretic peptides, and in other conditions, increased release of these hormones indicates cardiac stress or distress. For example, levels of brain natriuretic peptide, also called B-type natriuretic peptide (BNP), are elevated in systolic and diastolic dysfunction, ventricular hypertrophy and myocardial ischemia [64,65]. In cirrhotic patients, levels of atrial natriuretic peptide (ANP) and BNP are elevated due to increased cardiac release and not because of impaired hepatic extraction [66,67]. As BNP and its prohormone proBNP originate from ventricles, it is a more sensitive marker of ventricular dysfunction, especially in asymptomatic patients, than ANP which is released from atria. Increased circulating levels of BNP in asyptomatic cirrhotic patients are correlated to QT interval prolongation, interventricular septal thickness, left ventricular end-diastolic diameter and impairment of diastolic function [68,69]. Similarly, cardiac troponin I, a troponin isoform that is increased in myocardial injury, was reported to be elevated in patients with predominantly alcoholic cirrhosis [70]. At present, the exact role of these markers including natriuretic peptides and troponin isoforms in the noninvasive diagnosis of cirrhotic cardiomyopathy, remains to be fully clarified.

Some studies have used the measurement of systolic time intervals as a noninvasive tool to evaluate ventricular contractile performance in cirrhosis. The preejection period (PEP) to left ventricular ejection time (LVET) ratio is an important parameter for assessing left ventricular contractile function. In cardiomyopathy of all types, PEP is prolonged and LVET is shortened; thus, PEP/LVET is increased [71]. However, we believe that failure to stress the ventricle by a physiological or pharmacological challenge may not be able to detect the presence of this syndrome, because a hallmark of cirrhotic cardiomyopathy is the subnormal response to stressful stimuli. Some have advocated using dobutamine stress echocardiography [41] but a potential theoretical problem with this test is the downregulation of **β**-adrenergic receptors in the cirrhotic heart. Recently, Torregrosa *et al*. [36] studied cirrhotic patients using echocardiography and stress radionuclide ventriculography (using physical exercise by a graded bicycle ergometry in the same position, starting at zero workload and increasing by 10 W every 1 min). Some type of standardized exercise protocol appears to be an effective and safe stress challenge, but still the ideal screening test for cirrhotic cardiomyopathy remains unclear at present.

## Differential diagnosis

Many cardiac conditions resulting in mild or moderate left ventricular failure may mimic cirrhotic cardiomyopathy. Patients with liver disease may also have concomitant primary cardiac diseases such as ischemic heart disease, hypertensive cardiomyopathy or alcoholic cardiotoxicity. Whether patients with cirrhosis are somehow protected from developing coronary atherosclerosis remains unclear. Although this was formerly accepted as dogma, more recent evidence suggests that cirrhotic patients can indeed manifest severe coronary artery disease [72]. In Western countries, alcohol remains a significant cause of cirrhosis. In that regard, alcohol may precipitate or aggravate heart disease in two ways. First, it is well known that hypertension becomes more severe or uncontrolled in alcoholic patients (although if end-stage cirrhosis supervenes, then arterial pressure often normalizes due to peripheral vasodilatation). Second, chronic excessive alcohol is directly cardiotoxic, with alcoholic cardiomyopathy a well-described syndrome.

A careful assessment of patient history probing for excessive alcohol, physical examination for signs of hypertension such as retinal vascular changes, and appropriate diagnostic tests such as exercise stress electrocardiography, nuclear heart scans and coronary angiography will help distinguish these conditions from cirrhotic cardiomyopathy.

## Prognosis

The exact prognosis of cirrhotic cardiomyopathy remains unclear. The weight of evidence indicates that the clinical features of the syndrome become more prominent or worsen in parallel with progression of liver failure [reviewed in [21,22]. In other words, the extent of cirrhotic cardiomyopathy generally correlates to the degree of liver insufficiency. In prognostic terms, as the two conditions are related, mortality and morbidity due to cirrhotic cardiomyopathy or its complicating effect on an intervention such as TIPS insertion, is difficult to pinpoint as solely due to cirrhotic cardiomyopathy per se. Such an example is provided by Cazzaniga and colleagues [47], who found on univariate analysis that the MELD score, an index of liver failure (Model End Stage Liver Disease), and diastolic dysfunction as manifested by the E/A ratio were significantly predictive of death following TIPS insertion. However, as noted previously, on multivariate analysis, only the day-28 E/A ratio was an independent predictor, thus suggesting the interrelationship between the E/A ratio and the MELD score.

## Management

The optimum management of cirrhotic cardiomyopathy has not been established since there are neither "gold standard" diagnostic criteria nor specific treatments at present, recognition of cirrhotic cardiomyopathy depends on a high level of awareness for the presence of this syndrome, particularly in patients with advanced cirrhosis who undergo significant surgical, pharmacological or physiological stresses.

If heart failure becomes overt, the usual treatments should be initiated as for all forms of congestive heart failure, including bedrest, oxygen, diuretics and careful preload reduction by drugs [7,73].

Because the **β**-adrenergic receptor is desensitized in cirrhotic cardiomyopathy, inotropic **β**-agonists such as isoproterenol and dobutamine may not benefit patients with cirrhotic cardiomyopathy. In this regard, previous studies have demonstrated markedly attenuated vascular responses to isoproterenol and dobutamine in cirrhotic patients without overt evidence of heart failure [23,74].

There remains a significant paucity of human or animal data about specific pharmacological treatments [73,75]. Specific therapies such as **β**-blockade remain generally untested in humans or animals with cirrhosis. Henriksen and colleagues have reported that acute administration of a single dose of the **β**-blocker propranolol improves the prolonged electrocardiographic QT interval in cirrhotic patients [76], but whether there is also an improvement in contractile dysfunction with chronic dosing remains unknown. Cardiac glycoside therapy may not be beneficial; the short-acting glycoside ouabain was ineffective in patients with severe alcoholic cirrhosis in a study that dates back 3 decades [10]. Much future research, both clinical and experimental, is needed in this area. Pozzi and colleagues [77] recently reported that 6 months of treatment with the aldosterone antagonist canrenoate partially remodeled the hypertrophied ventricle in cirrhotic patients, *i.e*., reduced left ventricular chamber sizes and wall thickness. However, while diastolic function improved slightly, these did not reach statistical significance. The authors suggested, quite reasonably, that a more prolonged course of treatment might have allowed such changes to reach significance. In any case, angiotensin/aldsterone inhibitors appear to be a very promising potential treatment for cirrhotic cardiomyopathy and need further study.

Orthotopic liver transplantation (OLT) remains the gold standard of curative therapy in most liver diseases. Even though the immediate perioperative stresses, particularly increased arterial pressure (thus increased afterload) associated with this operation may unmask latent or overt myocardial dysfunction [78,79], yet with time, adaptation to the increased afterload occurs. Hence cardiac function gradually improves. As mentioned before, the prolonged QT interval generally normalizes after liver transplantation in most patients. More importantly, Torregrosa1 *et al*. [36] in a prospective study reported that OLT caused significant improvement of heart function parameters. In fact, virtually all cardiac alterations detected before transplantation returned to normal: the hyperdynamic state disappeared, left ventricular wall thickness and left atrial diameter also decreased. Both systolic and diastolic function normalized [36]. These results need to be confirmed in further studies but for now, strongly suggest that liver transplantation reverses cirrhotic cardiomyopathy [80].

## Unresolved questions

Consensus definition criteria, specific diagnostic tests, the exact prevalence of this syndrome and appropriate management strategies are all unresolved questions requiring further study.

## Conclusion

Cirrhotic cardiomyopathy is the term used to describe a constellation of features indicative of abnormal heart structure and function in patients with cirrhosis. These include systolic and diastolic dysfunction, electrophysiological changes and macroscopic and microscopic structural changes. The functional changes often require a significant stress challenge (exercise or pharmacological) to become manifest. To date, there is no single diagnostic test that can identify patients with this condition. Reversibility is possible (either pharmacological or after liver transplantation), but further studies are needed.
